# Resolving Power and Collision Cross Section Measurement Accuracy of a Prototype High-Resolution Ion Mobility Platform Incorporating Structures for Lossless Ion Manipulation

**DOI:** 10.1021/jasms.1c00056

**Published:** 2021-03-18

**Authors:** Jody C. May, Katrina L. Leaptrot, Bailey S. Rose, Kelly L. Wormwood Moser, Liulin Deng, Laura Maxon, Daniel DeBord, John A. McLean

**Affiliations:** † Center for Innovative Technology, Department of Chemistry, Vanderbilt Institute of Chemical Biology, Vanderbilt Institute for Integrative Biosystems Research and Education, Vanderbilt-Ingram Cancer Center, Vanderbilt University, Nashville, Tenessee 37235, United States; ‡ MOBILion Systems, Chadds Ford, Pennsylvania 19317, United States

## Abstract

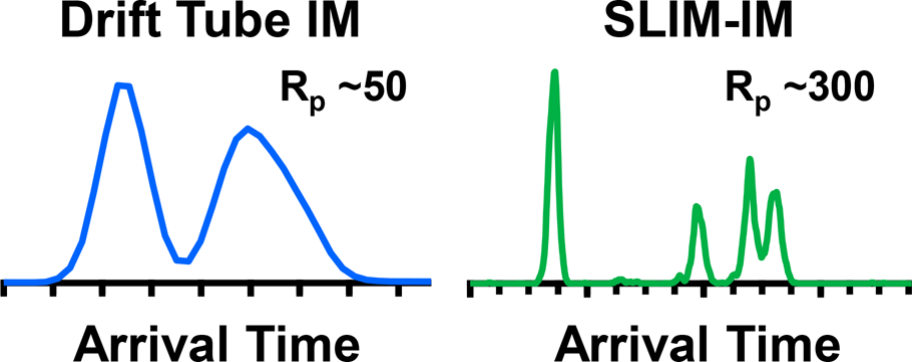

A production prototype structures for lossless ion manipulation ion mobility (SLIM IM) platform interfaced to a commercial high-resolution mass spectrometer (MS) is described. The SLIM IM implements the traveling wave ion mobility technique across a ∼13m path length for high-resolution IM (HRIM) separations. The resolving power (CCS/ΔCCS) of the SLIM IM stage was benchmarked across various parameters (traveling wave speeds, amplitudes, and waveforms), and results indicated that resolving powers in excess of 200 can be accessed for a broad range of masses. For several cases, resolving powers greater than 300 were achieved, notably under wave conditions where ions transition from a nonselective “surfing” motion to a mobility-selective ion drift, that corresponded to ion speeds approximately 30–70% of the traveling wave speed. The separation capabilities were evaluated on a series of isomeric and isobaric compounds that cannot be resolved by MS alone, including reversed-sequence peptides (SDGRG and GRGDS), triglyceride double-bond positional isomers (TG 3, 6, 9 and TG 6, 9, 12), trisaccharides (melezitose, raffinose, isomaltotriose, and maltotriose), and ganglioside lipids (GD1b and GD1a). The SLIM IM platform resolved the corresponding isomeric mixtures, which were unresolvable using the standard resolution of a drift-tube instrument (∼50). In general, the SLIM IM-MS platform is capable of resolving peaks separated by as little as ∼0.6% without the need to target a specific separation window or drift time. Low CCS measurement biases <0.5% were obtained under high resolving power conditions. Importantly, all the analytes surveyed are able to access high-resolution conditions (>200), demonstrating that this instrument is well-suited for broadband HRIM separations important in global untargeted applications.

## Introduction

Ion mobility (IM) has emerged as a robust separation strategy for complex chemical analyses largely due to its ability to be interfaced with mass spectrometry (MS) to improve peak capacity and aid in the separation of isobaric signals. Whereas conventional ion mobility does not perform at the same level of selectivity and resolution as liquid and gas chromatography (LC and GC, respectively),^1^ IM separations are several orders of magnitude faster than LC and GC (hundreds of milliseconds versus minutes) and can be integrated online with chromatography or MS imaging to further increase the peak capacity.^2,3^ Additionally, IM provides an additional molecular measurement, namely the gas-phase collision cross section (CCS), that can be used to gain further structural insight and support compound identifications.^4−6^ Although many of the technical hurdles associated with integrating IM with MS have largely been addressed, one of the contemporary challenges of IM has been the limited resolution offered by the technique. For example, MS routinely operates with resolving power (*R*_p_) values on the order of tens of thousands (time-of-flight MS) to hundreds of thousands (Fourier Transform MS, FTMS), yet the resolving power of current commercially available time-dispersive IM techniques is generally benchmarked below 100.^2,7−9^ Recent and notable exceptions do exist, namely trapped ion mobility spectrometry (TIMS), which demonstrates *R*_p_ values as high as 400,^10,11^ and a recently introduced cyclic multipass instrument based on traveling wave ion mobility spectrometry (TWIMS), which is capable of very high *R*_p_ values around 750 for 100 transits (∼1 m/transit) around the drift ring.^12^ These IM techniques typically require long scan periods (e.g., ∼1.5 s for 100 passes in cyclic TWIMS),^12^ or target a narrow range of mobilities to access high resolutions, which is analogous to the resolution and throughput trade-offs for FTMS instruments, where the resolving power scales with the acquisition duration.

In 2014, Smith and co-workers developed a generalized ion optical architecture they termed structures for lossless ion manipulation (SLIM),^13−15^ which utilizes two-dimensional arrays of electrodes patterned on printed circuit boards (PCBs) that are driven by combinations of dynamic (RF) and static (DC) electrical potentials to allow ion populations to be trapped and accumulated,^16,17^ turned at right angles,^18,19^ selected through tee-junctions,^20,11^ and lifted (elevators and escalators) to different SLIM levels.^21,22^ Ion mobility separations in SLIM devices have been demonstrated with both traditional uniform electric fields (drift-tube ion mobility spectrometry, DTIMS),^65−23,14^ and dynamically switched potentials (TWIMS),^24−26^ the latter of which allows for ion transfer across long distances without utilizing high electrical potentials.^27,22^ Using several right-angle turns, a serpentine path SLIM device operated with traveling waves was demonstrated,^19^ and an extended ∼13 m path length IM spectrometer was subsequently developed^28,29^ that was capable of accessing resolving powers in excess of 300.^21,30^ A multipass design was later developed, enabling variable path lengths through multiple transits through the device, e.g., ∼1094 m via 81 passes, yielding averaged resolving powers in CCS space of ∼1860.^31−67^ At the time of writing, this represents the highest resolving power published to date. Other SLIM technology developments in ion mobility have included filtering ions based on their mobilities,^14,33^ accumulating or compressing spatially dispersed ion packets (so-called CRIMP),^34,35^ and dual-polarity ion confinement and ion–ion reactions.^36−38^

In this work, we evaluate the separation capabilities of a preproduction prototype SLIM-based ion mobility spectrometer (SLIM IM) that utilizes the extended (∼13 m) separation path length design and operates with traveling waves to enable high-resolution ion mobility (HRIM) separations prior to mass analysis. In contrast to the conventional square wave operation of traveling wave IM, we evaluate the effects of both square and sine waveforms on the separation performance of this platform to provide performance metrics and guidance to the broader scientific community. The resolving power of this instrument is benchmarked in CCS space, and the separation capabilities are assessed using several isomeric systems. Finally, we develop a calibration method for converting SLIM IM arrival times to CCS and assess the CCS measurement bias of the instrument using a broadly available MS tuning mixture.

## Experimental Methods

### Sample Preparation

Agilent ESI-L Low Concentration Tune Mix containing fluoroalkyl phosphazene calibrant ions was used as received from the vendor. Peptides (SDGRG and GRGDS) and trisaccharides (melezitose, raffinose, maltotriose, and isomaltotriose) were purchased from Millipore-Sigma (St. Louis, MO). Gangliosides (GD1_a_ d18:1–18:0 and GD1_b_ d18:1–18:0) were purchased from Matreya, LLC (State College, PA). Triglycerides (1,2,3-tri-γ-linolenoyl-*rac*-glycerol and 1,2,3-tri-α-linolenoyl-*rac*-glycerol) were purchased from Cayman Chemical (Ann Arbor, MI). A complete list of chemical standards and their sources can be found in the Supporting Information (Table S1). All chemical standards except the triglycerides were obtained as dry powders and prepared individually to final working solution concentrations of ∼10 μg/mL in 1:1 methanol/water for IM-MS analysis. The triglycerides were obtained as 10 mg/mL solutions in ethanol and further prepared at 20 μM in 1:1 methanol/isopropanol with 13 μM ammonium acetate. All solvents were obtained from Fisher Scientific (Optima grade, Hampton, NH).

### Instrumentation

**Figure 1:**
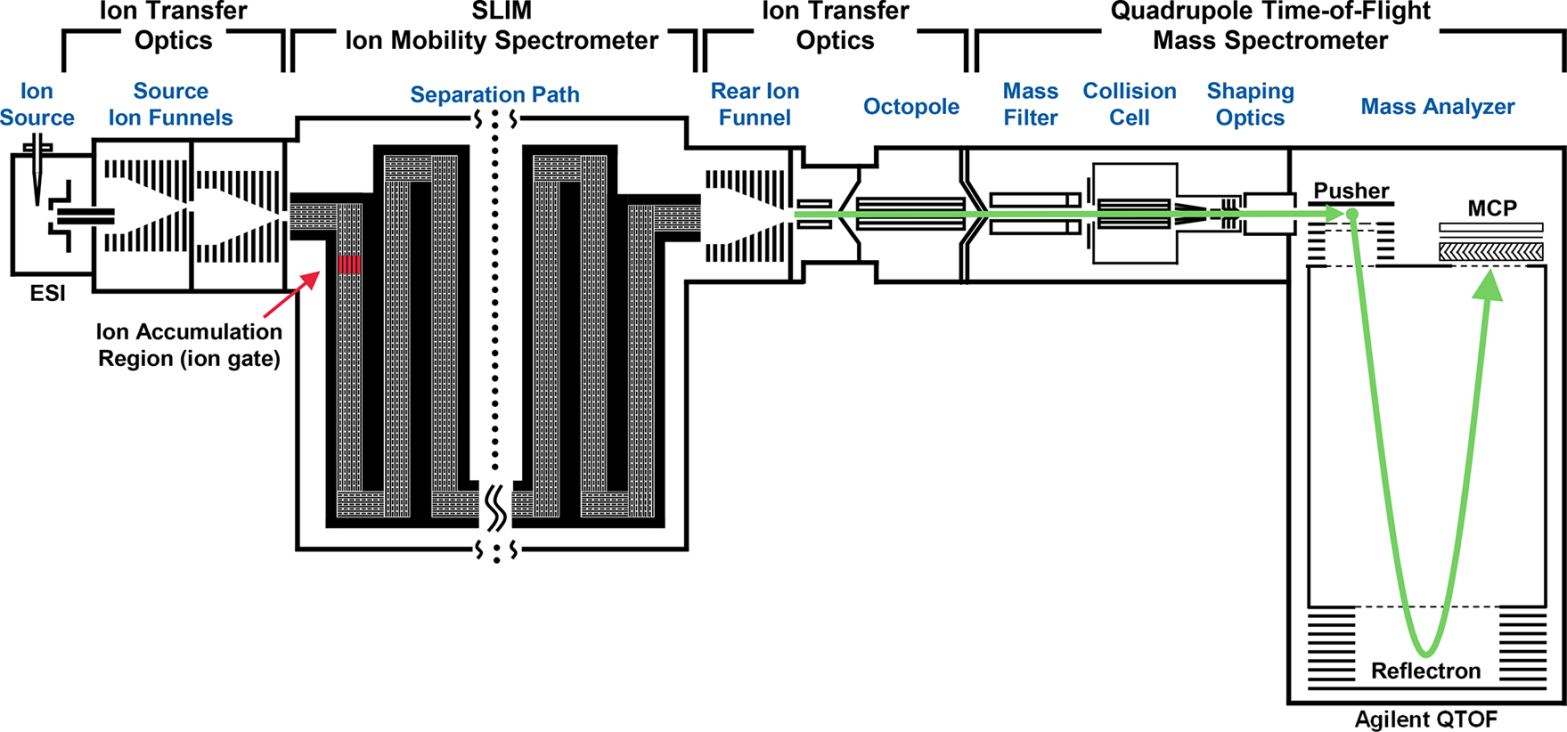
Schematic of the prototype SLIM IM-MS instrument used in this study, with significant components annotated.

Data were acquired using a prototype serpentine path SLIM ion mobility device (MOBILion Systems, Chadds Ford, PA) integrated with a commercial quadrupole time-of-flight mass spectrometer (6545, Agilent Technologies, Santa Clara, CA). A schematic of this IM-MS platform is shown in Figure 1. A liquid chromatography system (1290 Infinity II, Agilent) was used to introduce samples to the IM-MS via flow-injection analysis (20 μL of injection volume, 0.100 mL/min flow rate).^39^ Samples were ionized via electrospray (Jet Stream, Agilent) operated at 4.0 kV on the entrance capillary and −2.0 kV on the focusing nozzle lens. Ions were transferred to the vacuum via a resistive glass capillary and collected by two sequential source ion funnels (200 *V*_pp_, 1.1 MHz, 1–10 Torr), where they were focused radially and introduced to the SLIM PCB stack in which a set of SLIM electrodes were operated as an ion accumulation trap. The SLIM IM separation region utilized ∼2.5 Torr of high-purity nitrogen gas, which was metered by a gas flow controller (Alicat Scientific, Tucson, AZ) that was monitored with a capacitance gauge (627F Baratron, MKS Instruments, Andover, MA) and provided a regulation of better than ±0.002 Torr.

#### SLIM Ion Accumulation and Gating

**Figure 2:**
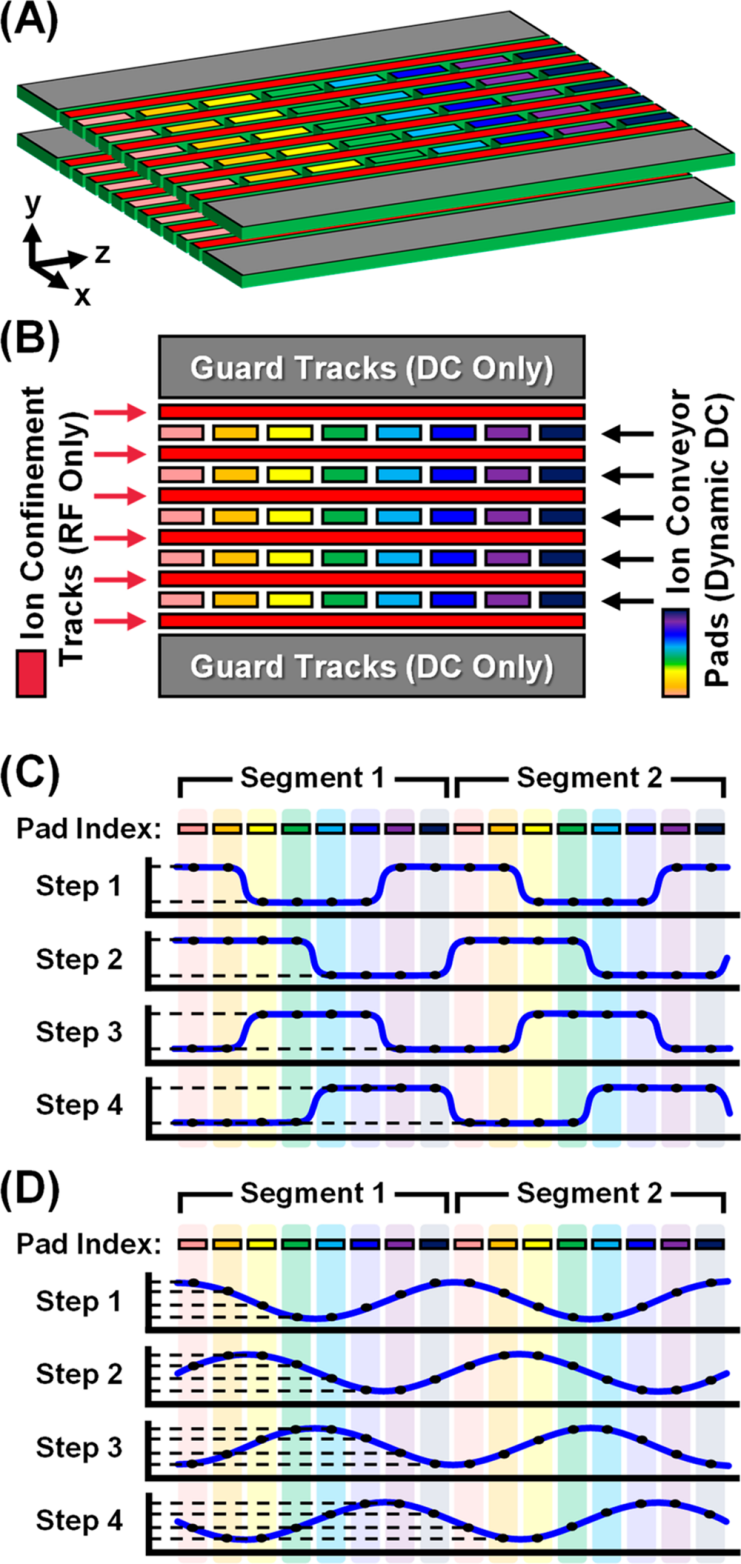
SLIM geometry and operational principles. (A) Three-dimensional representation of the printed circuit board stacking in the SLIM device for a short section incorporating one complete segment of the TW waveform (eight switching electrodes). (B) SLIM electrode layout. (C) Longitudinal wave propagation, square wave operation, and (D) sine wave operation. Ion conveyor-pad electrodes are grouped into sets of eight longitudinal electrodes. At each sequential time point, the wave progresses along the track, propelling ions along the traveling wave at average velocities related to their gas-phase mobilities.

Ion gating was achieved in the “ion accumulation region” within the SLIM device (Figure 1 callout), which operated a segment of the SLIM as a store-and-release ion trap by applying a repulsive DC potential (±80 V relative to the SLIM DC bias in the positive or negative ion mode) for 10 ms (ion accumulation time), and then restoring this potential to the dynamic traveling wave for ion release.^25^ This trapping potential was applied to the last set of dynamic DC electrodes in the segment (corresponding to the last column of dark blue pads in Figure 2).

#### SLIM Separation Path

The SLIM electrode geometry comprising the IM region is based upon a previous design,^21,25^ which utilizes a 13 m serpentine ion path length with 44 U-shaped turns (Figure 1, Separation Path). In SLIM, two planar boards with mirrored electrode geometries are stacked (∼3 mm apart) to establish the fields necessary for ion manipulation (Figure 2A). As previously described,^29^ the SLIM surface-electrode design on each board consists of the following three electrode types: a pair of outer guard tracks (3 mm width, 15 V) with DC-only potentials; six rows of inner RF-only tracks (∼0.5 mm width, 300 *V*_pp_, 735 kHz) for ion confinement; and five rows of ion conveyor pads (∼0.5 mm width by ∼1.0 mm length), which establish the traveling wave potentials used for ion manipulation. In this work, eight conveyor pads in each row (a set) are used to establish one cycle of either a square or a sinusoidal waveform by applying different potentials to each pad. A digitally generated waveform is applied to each individual segment of electrodes, with the adjacent electrodes receiving the same waveform but shifted by 45°. A total of >1400 sets (nearly 60 000 pad electrodes) were used across the 13 m serpentine path. With a pad-to-pad distance of 1.125 mm (1.0 mm pad length, 0.125 mm gap between pads) and the traveling wave stepped across eight pads (9.0 mm total length) to complete a single phase of the waveform, the wave switching frequencies surveyed in this work (5–25 kHz) correspond to wave speeds between 45 and 225 m/s (Table S1). Both the wave speed and the peak-to-peak wave amplitude (30–40 *V*_pp_) directly influence the ion mobility dispersion observed for traveling wave operation. These parameters are the same as the wave velocity and the wave height, respectively, which are commonly used to tune TWIMS instruments.

Following the IM separation, ions exit the SLIM boards, are collected by a rear ion funnel (200 *V*_pp_, 1.1 MHz, 2.5 Torr), and are transferred through an exit quadrupole to the conventional front optics of the Q-TOF for mass analysis. While this MS platform incorporates quadrupole-selective tandem MS/MS capabilities, for the present studies these elements are operated in a “pass-through” mode. SLIM IM-MS data were acquired using an 8-bit ADC digitizer (U1084A, Keysight Technologies).

### Software

Q-TOF instrument control and MS data acquisition was accomplished via the MassHunter data acquisition software (ver. 9.00, Agilent). The SLIM IM module control and data acquisition utilized a custom user interface (GAA Custom Engineering). IM-MS data were viewed and processed using MassHunter IM-MS Browser (ver. 10). IM traces were integrated across narrow *m*/*z* ranges and imported into Excel (Microsoft) for further analysis, including peak fitting and peak metric calculations (arrival time centroid, resolution, resolving power, percent valley, etc.). IM profile data acquired for the assessment of the resolving power across various SLIM IM conditions were extracted using IM-MS Browser and further processed and visualized in the R statistical computing programming environment (R Core Team, Vienna, Austria) using the tidyverse suite of tools.^40^

### Resolution Calculations

IM resolution is commonly assessed using the “resolving power”, which is derived from measurements of a single peak in the IM dimension.^41^ To obtain resolving power values, IM arrival time data were converted to CCS space.^26^ Briefly, the peak centroids (*t*_p_) and peak widths calculated at the full-width at half-maximum height (Δ*t*_fwhm_) were obtained by applying normal distribution fits to the peak of interest within the extracted SLIM IM data traces. CCS values corresponding to these peaks were obtained in a separate experiment using a drift-tube instrument (6560 IM-QTOF, Agilent) as previously described.^42,43^ The known CCS values of two peaks (CCS_p1_, CCS_p2_) within the same drift spectrum were then used to obtain a CCS difference between two peaks (p1 and p2) as follows:
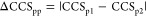
1A time difference between the two peaks, *Δt*_pp_, was similarly calculated from the time centroids of the peaks, *t*_p1_ and *t*_p2_ as follows:
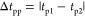
2This relationship was then used to convert the fwhm values to CCS space, which was used to calculate the CCS-based resolving power, *R*_p_(CCS/ΔCCS), as follows:
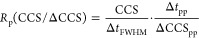
3This CCS-based resolving power is more representative of the separation capabilities for SLIM IM and can be used to make direct comparisons between the separation capabilities of different ion mobility techniques.^26,28^ It is noted here that this arrival time to the CCS conversion assumes a linear correspondence, whereas the relationship between TWIMS arrival times and CCS are known to be nonlinear;^44^ however the uncertainty is expected to be low due to the fact that the conversion is only applied across the width of a single peak. The average CCS is used when reporting the resolving power from a spectrum containing multiple peaks. Two-peak resolution (*R*_pp_) and percent valley (*V*) calculations were conducted as previously described.^45^

### CCS Calibration

High-precision CCS values for the eight tune mix ions were previously measured from a reference DTIMS instrument operated in nitrogen drift gas (^DT^CCS_N2_).^42^ These reference CCS values (CCS_ref_) were converted to “reduced CCS” (CCS′) by including the ion-neutral reduced mass (μ) and ion charge-state (*z*) dependencies (eq 4) as previously described.^46^
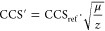
4Note here that CCS′ is different from the reduced CCS used in the ion transport community to rescale the CCS without hard sphere contributions.^47^ The tune mix ion raw arrival times were measured at various wave speeds and amplitudes, and plots of the reduced CCS versus the SLIM IM arrival time were fitted with nonlinear regression models using the R statistical programming environment. These models were then used as calibration equations to calculate the CCS of the tune mix ions from the SLIM IM measurements (^TW(SLIM)^CCS_N2_). As the accuracy of any given CCS measurement is unknown, the percent CCS bias between the calculated and reference DTIMS CCS values was used to assess the performance of the various models as follows:
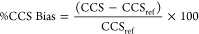
5The consensus CCS reference values used here have a reported interlaboratory repeatability of 0.22%;^43^ however, expanded uncertainty analysis has estimated that the uncertainty in these drift-tube CCS measurements falls within the range from 2.7 to 4.6%,^48^ thus limiting the CCS accuracy that can be obtained from this assessment.

## Results and Discussion

### Accessible Resolving Powers

Benchmarking experiments were conducted to determine the SLIM IM conditions in which the highest resolving powers were accessed. For these experiments, two waveforms (square and sine waves), five wave speeds (45, 90, 135, 180, and 225 m/s), and three wave amplitudes (30, 35, and 40 *V*_pp_) were evaluated, spanning the optimal transmission and separation ranges of the instrument. IM profile data were extracted for each of the eight tune mix ions that appear prominently in the positive ion mode (*m*/*z* 622, 922, 1222, 1522, 1822, 2122, 2422, and 2722). All data were acquired in triplicate, resulting in a total of *N* = 720 *R*_p_(CCS) values for the entire data set. Note that the high RF potentials (300 *V*_pp_) applied to the RF electrodes optimized the total ion signal but at a cost of a reduced low *m*/*z* transmission such that *m*/*z* 322 did not appear in high abundance and was thus not included in the analysis.

An initial assessment of the two waveforms was conducted, which indicated that both waveforms access similar resolving powers; however, the square wave data set exhibited a narrower optimal range of arrival times, which correspond to the highest *R*_p_(CCS) values (Figure S3). Additionally, fewer ions were observed to separate within the square wave data (*N* = 188, or 52% of the total data set) as compared to the sine wave data (*N* = 247, 69%), and recent work from Smith and co-workers has indicated that the square wave operation of SLIM IM can contribute to more ion heating than the sine wave operation.^49^ As such, only the sine wave data were evaluated in subsequent experiments. Considering that nearly all traveling wave IM operation to date has utilized a square waveform to approximate a sinusoidal axial potential,^23,50^ the broader-scale IM separation range observed when operating with a true sine wave in this work implies that the traveling wave technique can be improved using tailored waveforms. The full workup for the square wave data can be found in Figure S4. Hereafter, only the sine wave operation of the SLIM IM system will be discussed.

**Figure 3:**
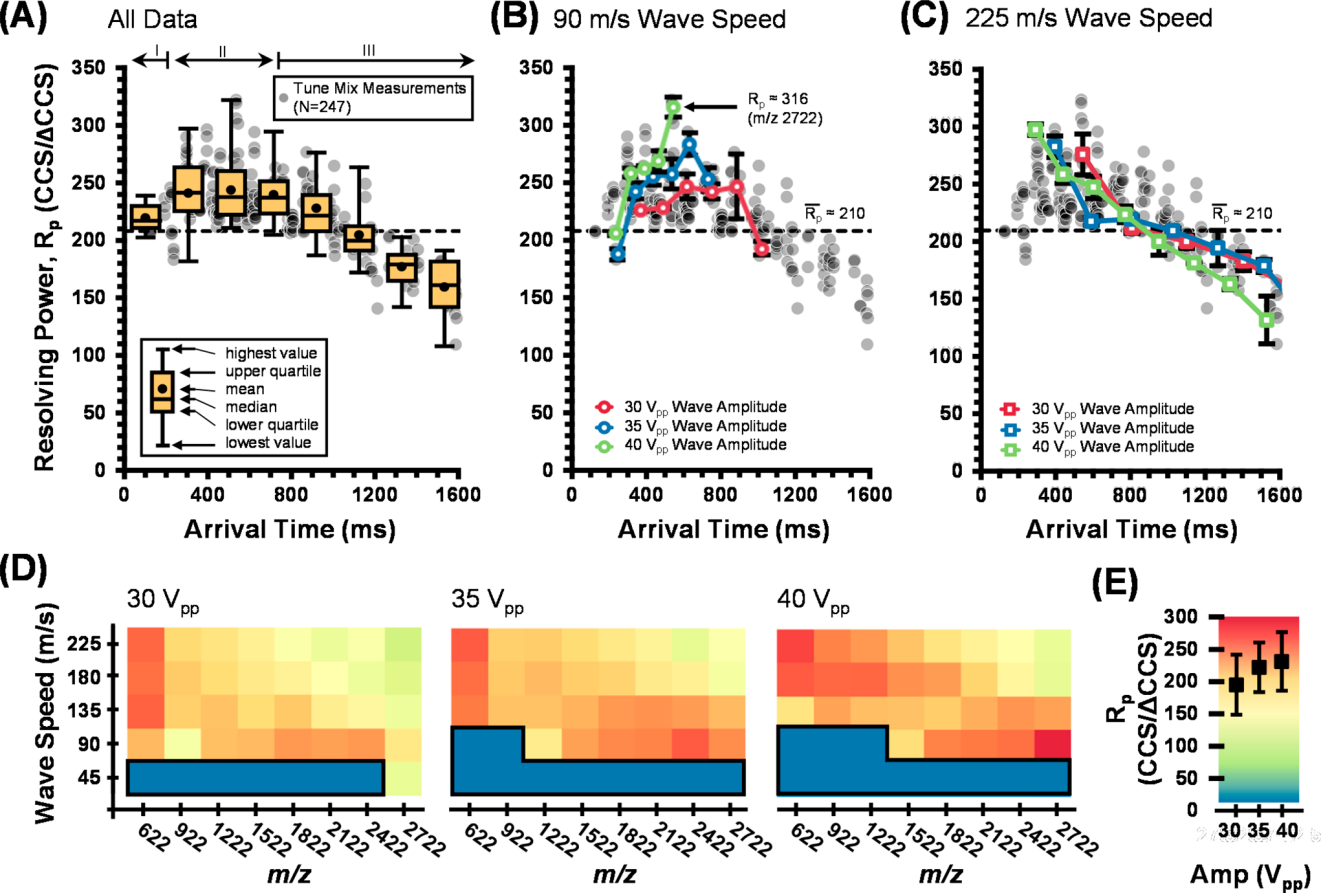
Visualization of the resolving power performance for a sinusoidal traveling wave across the various operational parameters explored in this study. (A) A summary of all the CCS-based *R*_p_ values (*N* = 247) as a function of the arrival time for tune mix components (cyclophosphazenes) measured across various SLIM IM parameters, including wave speeds (45, 90, 135, 180, and 225 m/s) and wave amplitudes (30, 35, and 40 *V*_pp_). The box and whisker overlays summarize the data within 200 ms bins. (B) Average *R*_p_(CCS) values (three replicates per data point) calculated for a 90 m/s wave speed at 30, 35, and 40 *V*_pp_ wave amplitudes. (C) Average *R*_p_(CCS) values calculated for 225 m/s data. The horizontal dotted line in these scatter plots represents the average *R*_p_ value across the entire data set (ca. 210). (D) Heat maps visualizing the resolving powers observed for each tune mix component (*x*-axis) at each wave speed (*y*-axis) for wave amplitudes of 30 (left panel), 35 (middle panel), and 40 *V*_pp_ (right panel). Each square represents an average of three replicate measurements. Here, the dark blue regions represent conditions in which ions are transmitted but no IM separation occurs (i.e., ion “surfing” conditions), which is observed at a low *m*/*z*, a high wave amplitude, and low wave speeds. (E) Color scale for panel D with average and standard deviation *R*_p_(CCS) values measured for the tune mix components at each wave amplitude (*x*-axis).

Results are summarized in Figure 3 for the optimization of the resolving power as a function of the sine wave speed, sine wave amplitude, and analyte mass-to-charge ratio. Only ions that exhibit mobility selective behavior (i.e., not surfing) are considered. Here and elsewhere, the raw arrival times are discussed, and it is important to note that the majority of this measured arrival time represents time spent within the SLIM path (i.e., the true IM drift time); however, there are contributions from the time spent in other portions of the instrument, including the ion transfer optics to the TOF stage. The scatter plot in Figure 3A reveals three distinct ranges of arrival times where different resolving power values are accessed.^51^ (I) For fast arrival times (<200 ms), the so-called “ion surfing” conditions, ion mobilities are too fast to allow IM-selective “roll-over” events to occur. Here, little to no IM separation occurs under most SLIM IM conditions (see Figure S2). (II) For intermediate arrival times between ca. 200 and 700 ms, the highest resolving powers were observed. In this region of ion motion, the ions are fully subject to mobility-selective ion drift throughout the SLIM separation path. The corresponding box-and-whisker plot in this range of arrival times shows that the majority of *R*_p_(CCS) values are between ca. 230 and 260 (242 mean value for the 400–600 ms bin), with a few data points exhibiting resolving powers in excess of 300. (III) For the slower arrival times beyond ca. 700 ms, the *R*_p_(CCS) magnitude gradually declines, which is interpreted as a result of peak broadening due to extended ion-gas diffusion that corresponds to long residence times within the SLIM IM separation path. The box-and-whisker plots at these long arrival times indicate that the majority of resolving powers continue to decline, falling below 200 for times greater than ∼1.2 s. Panels B and C in Figure 3 contain overlays of the average *R*_p_(CCS) values observed at each of the three wave amplitudes for low and high wave speeds (90 and 225 m/s, respectively). Here, the two wave speeds investigated represent conditions where ions were either undergoing transitions from ion surfing to IM-selective drift (Figure 3B, 90 m/s) or all experiencing continuous ion drift (Figure 3C, 225 m/s). The results are somewhat complicated, but in general the highest resolving power values were observed for ions near the transition from ion surfing to IM-selective drift (e.g., 90 m/s). At 90 m/s, the highest wave amplitude (40 *V*_pp_) generally accesses the highest resolving powers, which is consistent with previous TWIMS findings,^4^ although *R*_p_(CCS) differences no longer appear significant under conditions where all ions had fully transitioned to an IM-selective drifting behavior, e.g., region III (Figure 3C). Collectively, panels B and C in Figure 3 suggest that operating near the boundary of the IM-selective traveling wave behavior can offer a slight increase in the *R*_p_(CCS) value, specifically when operating at the highest wave amplitudes (40 *V*_pp_) under the lowest wave speeds that still yield IM separations (90 m/s). In other words, the highest resolving powers are obtained near the onset of ion surfing behavior, and this information can be used to target a high resolution for specific analyte systems. Under these conditions, ions are mobility-separated and spend the minimal amount of time within the elevated-pressure SLIM separation path, which otherwise leads to diffusional broadening of the peaks. Figure 3D recasts the resolving power data as a function of different *m*/*z* corresponding to the cyclophosphazene analytes. This projection is useful for illustrating the mass-dependent effects on the measured resolving powers. Each square within the heat map represents the *R*_p_(CCS) value (*N* = 3, averaged) for each tune mix ion measured at each wave speed (*y*-axis) and wave amplitude (panels) surveyed. The corresponding values for the heat map color scale and overlays of the average *R*_p_(CCS) values observed across each wave amplitude are contained in Figure 3E. Here, the ion-specific data confirm that the highest resolving powers were achieved for all ions at 35 and 40 *V*_pp_ wave amplitudes, although these projections also reveal that IM separation across the full range of masses surveyed was only achieved under the higher wave speeds above 135 m/s.

Of note is that the optimal conditions for the resolving power are slightly different for higher-mobility (lower *m*/*z*) species than those that were observed for lower-mobility (higher *m*/*z*) ions. When optimizing the overall resolving power of the SLIM IM, we recommend using the higher range of values for both wave speed (e.g., 180–225 m/s) and the wave amplitude (e.g., 35–40 *V*_pp_) to ensure all ions undergo mobility separation. However, to achieve the highest resolving power for a single species rather than a range of species, the highest wave amplitude (40 *V*_pp_) is recommended, and the wave speed can be adjusted to further optimize the separation (e.g., higher speeds for low *m*/*z* ions and lower speeds for high *m*/*z* ions). These results suggest that simultaneously scanning the wave speed and the wave amplitude should allow the highest *R*_p_ values to be achieved across a range of *m*/*z*, which is similar to the practice of operating TWIMS instruments using ramped wave heights.

Table 1 summarizes the highest *R*_p_ values observed and the corresponding SLIM IM conditions in which they were accessed for each tune mix ion. Overall, 40 *V*_pp_ wave amplitudes yielded the highest resolving powers. While in some cases the highest *R*_p_ value was observed at 35 *V*_pp_, for most of those occurrences the highest and second highest *R*_p_ values (Table S3) were within the reproducibility error of each other. Finally, the highest-mass tune mix component (*m*/*z* 2722) yielded the highest resolving powers at *R*_p_(CCS) = 316 for 40 *V*_pp_ and 90 m/s SLIM IM conditions. These observations hold for the phosphazenes, which are structurally stable compounds; however, more fragile ions may not be able to access the same level of resolving power due to different levels of ion heating experienced under the different wave amplitudes and speeds. It is also noted that these results are for singly charged ions—higher resolving powers are expected when investigating higher charge states (*vide infra* ganglioside results).

**Table 1:** Highest Resolving Power Values Observed

		corresponding parameter		
tune mix ion	highest *R*_p_ measured (CCS/ΔCCS)^a^	wave amplitude (*V*_pp_)	wave speed (m/s)	corresponding arrival time (ms)
*m*/*z* 622	297.4 ± 5.0 (3)	40	225	290.5
*m*/*z* 922	274.1 ± 16.6 (3)	40	180	437.9
*m*/*z* 1222	276.0 ± 22.5 (3)	40	180	462.9
*m*/*z* 1522	251.8 ± 13.2 (3)	40	180	596.4
*m*/*z* 1822	258.8 ± 3.7 (3)	40	90	304.7
*m*/*z* 2122	263.2 ± 1.5 (3)	40	90	381.6
*m*/*z* 2422	283.6 ± 9.5 (3)	35	90	622.1
*m*/*z* 2722	315.7 ± 8.6 (3)	40	90	535.4

a The highest *R*_p_ is averaged over the replicate measurements, the number of which is denoted in the parentheses. The time-to-CCS conversion was determined from eq 3 using the differences between the tune mix ion and the next-highest *m*/*z* ion in the spectrum.

### Collision Cross Section Calibration

Recent work from Smith and co-workers evaluated the use of different negative-mode calibrants for use with a 13 m SLIM IM (SLIM “SUPER”),^49^ which has the same geometry as the platform used in this current study with the exception that the SLIM SUPER incorporates an additional return path to perform multipass experiments.^31^ In addition to evaluating the negative mode, their work assessed both sine and square wave operations under three wave amplitudes (40, 50, and 60 *V*_pp_) at a fixed wave speed (200 m/s).

**Figure 4:**
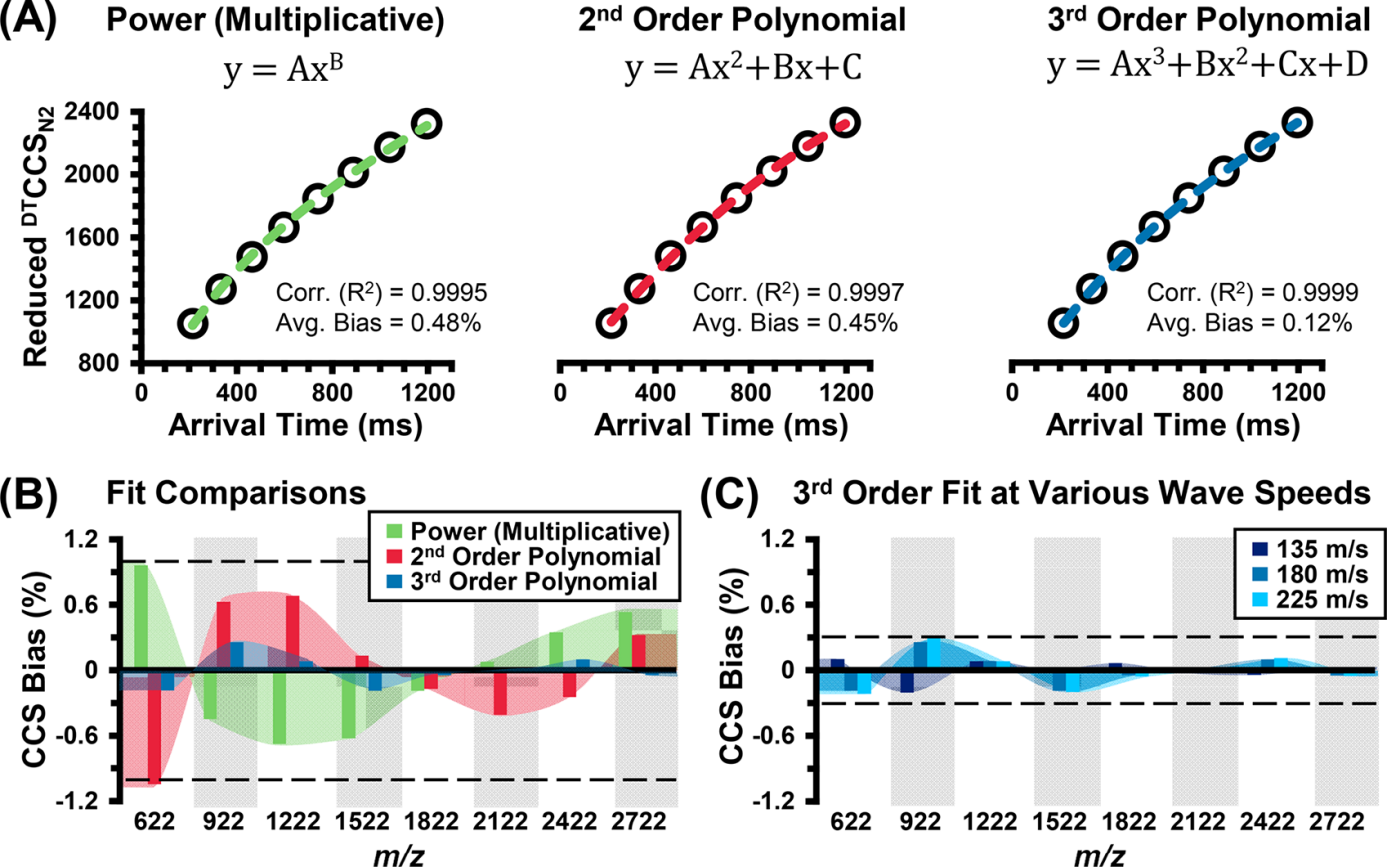
Influence of different calibration equations and instrument parameters on the calibration of SLIM IM arrival times of the tune mix ions to CCS. (A) The coefficients of determination (*R*^2^) and average biases across all ions for the following three fit equations: a power fit (left panel), a second-order polynomial (center panel), and a third-order polynomial (right panel). Data were obtained under mobility-selective conditions at 40 *V*_pp_ and 180 m/s. (B) Summary of the CCS biases associated with each fit equation. (C) CCS biases observed at 40 *V*_pp_ and different wave speeds. Each data point was measured in triplicate.

Here, we evaluate the positive ion mode and sine wave operation of the HRIM SLIM platform. Because the choice of the calibrant strongly affects the CCS calibration in TWIMS, we pragmatically chose to only assess tune mix to determine the parameters that yield the lowest CCS errors when other factors are not considered. Figure 4A contains plots of the reduced CCS of the reference values versus the SLIM IM arrival times, which are fitted with the following three calibration equations: a power fit, a second-order polynomial fit, and a third-order polynomial fit. Here, 40 *V*_pp_ and 180 m/s were chosen for this assessment, as they yielded the overall-highest resolving powers where none of the tune mix ions were surfing (Figure S5). Our results indicate that the CCS errors are the lowest when using a third-order polynomial fit (*R*^2^ = 0.9999), with an average CCS bias across all the ions of 0.12%. While the lower error for a third-order fit was also noted in the work from Smith and co-workers,^47^ here we observed a significant improvement when using a third-order polynomial compared to using a conventional power fit (*R*^2^ = 0.9995, 0.45% bias), which is summarized in Figure 4B. Using the third-order fit, we also evaluated three different wave speeds (135, 185, and 225 m/s) under which ions did not undergo surfing behavior; all three yielded similar CCS errors, with the lower wave speed, 135 m/s, exhibiting the lowest absolute CCS bias of 0.07%. Errors associated with the other SLIM IM wave speeds and amplitudes for the third-order polynomial fit are summarized in Figure S6.

For the CCS determination, we found that the SLIM IM conditions that yield the highest resolving powers are also best-suited for CCS calibration, namely, 40 *V*_pp_ and 135 m/s. Regarding the calibration equation itself, we note that all three fit equations yielded low errors of less than a 0.5% bias as compared to the reference CCS values and emphasize that polynomial fits cannot be extrapolated without a high error. Thus, a power fit is recommended when a generalizable calibration is desired, whereas the third-order polynomial fit can be used when the lowest CCS errors are needed and the CCS values fall within the range of calibration. Additional errors are expected when applying these calibrations to other ions; however, this evaluation serves to assess the errors associated with the calibration method itself as well as the lowest fit errors that can be expected with this approach, namely, less than 0.2%. Additional refinements to this calibration method, such as incorporating a time correction to the raw arrival time data or implementing a modified power law function, should further improve the error of this approach.

### Separation of Isomeric Mixtures

**Figure 5:**
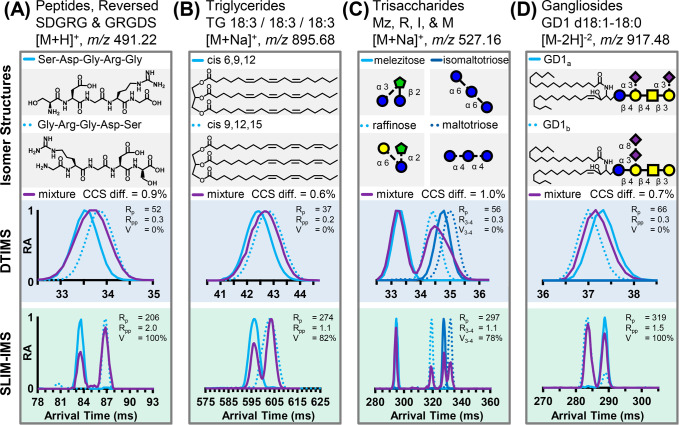
Comparative isomer separations of DTIMS and the prototype SLIM IM system. Chemical structures are shown in the gray boxes (top row). Standard-resolution DTIMS mobility traces are shown for the individual components and mixtures in the blue boxes (middle row). High-resolution SLIM IM mobility traces and associated separation metrics are shown for the individual components and mixtures in the green boxes (bottom row). (A) Reverse-sequence peptides. (B) Triglycerides exhibiting double-bond-position isomerism. (C) Trisaccharide isomers with various monosaccharide subunits and linkages. Here, the blue circle is glucose, the yellow circle is galactose, and the green pentagon is fructose. The CCS difference, *R*_pp_, and *V* are calculated between the third and fourth peaks. (D) Ganglioside glycosphingolipids with different sialic acid linkages at the headgroup. Here, the yellow square is *N*-acetyl-galactosamine, and the purple diamond is *N*-acetyl-neuraminidate. “RA” refers to the relative abundance, which is normalized to the most abundant feature within the spectrum.

Four isomer systems were selected to investigate the capability of the high-resolution SLIM IM platform to resolve challenging isomeric mixtures. These isomer systems, the standard and HRIM spectra, and the corresponding separation metrics are summarized in Figure 5 and discussed below. Individual traces for the drift-tube and SLIM IM measurements are contained in Figures S7 and S8, respectively. Corresponding CCS values needed for calculating the separation metrics were also measured on a drift-tube instrument and are summarized in Table S4. For all SLIM results except the triglycerides, IM spectra were obtained under conditions where the ions just began to transition to IM-selective drift through the SLIM IM separation path (180 m/s and 40 *V*_pp_).

First, a pair of reverse-sequence pentapeptides, SDGRG and GRGDS, were investigated (180 m/s and 30 *V*_pp_). The ion mobility separation of this system was first reported by Hill and co-workers for the doubly protonated ion form ([M + 2H]^2+^, *m*/*z* 246) using a drift tube operated at ambient pressure^52^ and is currently used extensively by the ion mobility community for benchmarking IM separation capabilities. Whereas numerous IM studies have demonstrated the full resolution of the doubly protonated ion forms of the SDGRG/GRGDS mixture,^53−55^ the singly protonated ion form ([M + H]^+^, *m*/*z* 491) is more challenging to resolve. The full-baseline resolution of this ion system has been demonstrated for TIMS,^56^ cyclic TWIMS,^12^ and a 13 m SLIM instrument^24^ similar to that used in this work. Notably, the cyclic TWIMS instrument developed by Giles and co-workers demonstrated the full resolution of this ion system after 4 passes, with reported *R*_p_(CCS/ΔCCS) values as high as 350 after 16 passes around the TWIMS ring.^12^ In this work, the singly protonated ion forms of SDGRG and GRGDS also exhibit full baseline resolution in the SLIM IM prototype, with IM elution orders consistent with those of the previous work (Figure 5A, bottom panel). Standard-resolution DTIMS measurements yielded CCS values of 203.5 and 205.4 Å^2^ for the protonated ion forms of SDGRG and GRGDS, respectively, which correspond to a CCS difference of ∼0.9%. As expected, the resolving power accessible from conventional DTIMS (∼50) is not sufficient to resolve these isomers (Figure 5A, middle panel); however, HRIM analysis provides baseline resolution at a calculated *R*_p_(CCS/ΔCCS) of ∼210.

Next, a pair of triglycerides (TG) possessing different double-bond positions (*cis* 6, 9, and 12 or *cis* 9, 12, and 15) were analyzed individually and as mixtures using both standard- and high-resolution IM (SLIM settings were 180 m/s and 40 *V*_pp_). Triglycerides are neutral lipids that exhibit a prominent ion signal in the positive mode corresponding to the adduction of a positive charge carrier (commonly an alkali metal, Na^+^ or K^+^, or ammonium, NH_4_^+^). Here, the sodiated ion forms ([M + Na]^+^, *m*/*z* 896) were investigated, which appear in high abundance in the spectra. The standard-resolution DTIMS results demonstrate similar CCS differences for the TGs as those observed for the reversed-sequence peptides; similarly, the TG isomer mixture was not resolved (Figure 5B, middle panel), although the DTIMS resolving power was relatively low (∼40). This lower-than-typical resolving power was also observed for the SLIM IM results, though here the near-baseline resolution of the isomer mixture was still achieved (*R*_pp_ = 1.1 and *V* = 82%) with a resolving power of 274. One possible explanation for the broader peaks observed in this system is that the IM spectra are comprised of unresolved features, though it is also important to note that the TG SLIM spectra were acquired within a batch of lipid mixtures using untargeted IM parameters tuned for broadband IM separations and consequently do not represent the most optimal resolution conditions. This TG isomer system has not been previously investigated by IM.

A set of four trisaccharide isomers (melezitose, raffinose, isomaltotriose, and maltotriose), representing various monosaccharide subunit types and linkages, were analyzed individually and as a mixture by both DTIMS and SLIM IM (SLIM settings were 225 m/s and 40 *V*_pp_). These isomers are routinely used by the IM community to benchmark separation performance.^57−62^ For carbohydrates, the sodiated ion form appears in a high abundance and is investigated here ([M + Na]^+^, *m*/*z* 527). For the mixture of isomers, melezitose exhibits the lowest CCS and appears far-removed from the other trisaccharides. The latter three isomers (raffinose, isomaltotriose, and maltotriose) all appear as a broad and unresolved distributions under standard resolution DTIMS analysis (Figure 5C, middle panel). The analysis of the individual isomer standards indicates that the isomaltotriose–maltotriose pair exhibits the closest spacing among these sugars with a CCS difference of 1.0% and thus are the most challenging of the set to resolve. These isomers differ only by the glycosidic linkages between each glucose subunit, which one might expect would not result in large differences in the CCS. HRIM analysis (Figure 5C, bottom panel) of the four-component trisaccharide mixture yielded full resolution of all but the last two isomers (peaks 3 and 4), which were resolved with a 78% valley (*R*_pp_ = 1.1). The corresponding resolving power is ca. 300, as averaged over the values determined from the individual isomer peaks. The elution order of this isomer system is consistent with results obtained for the individual standards as well as previous IM studies.

Finally, two ganglioside glycosphingolipid isomers (GD1_a_ and GD1_b_, 36-carbons) were investigated (SLIM settings were 180 m/s and 40 *V*_pp_). The IM separations of these gangliosides were previously reported using a relatively short 1.25 m SLIM device.^28^ While this prior study demonstrated baseline resolution for the doubly sodiated ion forms ([M + 2Na]^+2^, *m*/*z* 941) of the GD1 isomer mixture, DTIMS measurements indicate that these ion forms of GD1_a_ and GD1_b_ are well-separated in CCS space (CCS difference of 1.9%). More challenging to resolve are the doubly deprotonated ions ([M – 2H]^−2^, *m*/*z* 917) of the GD1_a_/GD1_b_ isomer mixture, which are observed prominently in the negative ion mode and exhibit a CCS difference of only 0.7%. Negative ions are highly relevant in lipidomics research as polar lipids often dominate the positive ion mode spectra, and many lipid types are also only detectable in negative ion mode.^63,64^ While the standard-resolution DTIMS spectra did not resolve the GD1 mixture (Figure 5D, middle panel), the HRIM spectra (bottom panel) show both lipids are baseline-resolved in a mixture (*R*_pp_ = 1.5, 100% valley). Here, the corresponding GD1 peaks exhibit CCS differences of less than 1%, which are otherwise very challenging to separate. In this particular example, the primary GD1 ion forms investigated ([M – 2H]^−2^) are doubly charged, which benefits IM separation as higher charge-state ions are capable of accessing higher resolutions in ion mobility, here >60 for standard-resolution DTIMS and >300 for HRIM analysis using SLIM IM.

## Conclusions

The accessible resolution and CCS measurement capabilities of a SLIM-based HRIM-MS system were critically evaluated. This preproduction prototype instrument is based directly on a previous IM-MS design and similarly utilizes structures for lossless ion manipulation to enable the transfer and mobility separation of ions across a large distance (∼13 m) for HRIM analyses. The resolving power (CCS/ΔCCS) of the SLIM IM device was benchmarked to between 230 and 315 for a commonly used MS tuning mixture, corresponding to the highest wave amplitudes surveyed in this study (35 and 40 *V*_pp_). The optimal resolving powers were observed under conditions where ion arrival times were between 1.5 and 3× the arrival times associated with surfing-only behavior, which corresponds to ion speeds that are 30–70% of the speed of traveling wave itself. Notably, all the ions from the mixture were transmitted within a short dispersion time frame (<700 ms) and were able to access CCS-based resolving powers in excess of 230, suggesting that this IM-MS platform is well-suited for broadband untargeted studies. For the targeted separations of several biochemical isomers (peptides, lipids, and carbohydrates), the HRIM-MS platform achieved near- or full-baseline resolutions for the corresponding isomeric mixtures and measured peak spacings with as little as a 0.6% difference in CCS.

## Supporting Information Available

Information on the chemical standards used and their sources, corresponding wave speeds for SLIM IM wave-switching frequencies, tabulated conditions where the second-highest resolving powers were observed, CCS measurements obtained on the drift tube instrument (^DT^CCS_N2_), the square versus sine wave operation, the IM-MS spectrum observed under ion surfing conditions, average resolving powers, CCS calibration biases, and individual ion mobility spectra (PDF)
