# Supplementary material for: Resolving Power and Collision Cross Section Measurement Accuracy of a Prototype High-Resolution Ion Mobility Platform Incorporating Structures for Lossless Ion Manipulation
Source: J Am Soc Mass Spectrom. 2021 Mar 18;32(4):1126–37. doi: 10.1021/jasms.1c00056 (PMC9296130; doi:10.1021/jasms.1c00056)
Supplement: Supplementary file 1 — js1c00056_si_001.pdf [file js1c00056_si_001.pdf]

Supporting Information

Resolving Power and Collision Cross Section Measurement Accuracy of a Prototype High Resolution Ion Mobility Platform Incorporating Structures for Lossless Ion Manipulation

Jody C. May[†], Katrina L. Leaptrot[†], Bailey S. Rose[†], Kelly L. Wormwood Moser[‡], Liulin Deng[‡], Laura Maxon[‡], Daniel DeBord[‡], and John A. McLean[*,†]

[†]Center for Innovative Technology, Department of Chemistry, Vanderbilt Institute of Chemical Biology, Vanderbilt Institute for Integrative Biosystems Research and Education, Vanderbilt-Ingram Cancer Center, Vanderbilt University, Nashville, TN, 37235, United States

[‡]MOBILion Systems, Chadds Ford, Pennsylvania 19317, United States

[*] Corresponding Author Email: john.a.mclean@vanderbilt.edu

SI Contents:

Table S1. – Information on chemical standards and their sources…............................ S2
Table S2. – Corresponding wave speeds for wave switching frequencies.…………………..…S3
Table S3. – Second-highest resolving powers observed…..……………………………….…. S4
Table S4. – Drift tube ion mobility (DTIM) collision cross section measurements………..…S5

Figure S1. – Square versus sine wave operation………...……………….………….………... S6
Figure S2. – IM-MS spectrum showing ion surfing behavior …..........………...……….……. S7
Figure S3. – Averaged resolving powers observed for sine and square wave.……………… S8
Figure S4. – Resolving power performance of square wave operation.…...……………….... S9
Figure S5. – Resolving power plots for all conditions surveyed…………………………….... S10
Figure S6. – CCS calibration biases observed for all conditions………...………………….... S11
Figure S7. – DTIMS spectra of isomers, individual and mixtures…………..………………....S12
Figure S8. – SLIM IM spectra of isomers, individual and mixtures……….....……………….. S13

Table S1. Chemical Standards Information

Analyte Abbreviation	Full Name	Vendor Source (Product Number)	CAS Registry Number
HP-321	hexamethoxyphosphazene	Agilent Technologies (ESI-L, G1969-85000)	957-13-1
HP-621	hexakis(2,2-difluoroethoxy) phosphazene		186817-57-2
HP-921	hexakis(2,2,3,3-tetrafluoropropoxy) phosphazene		58943-98-9
HP-1221	hexakis(1h,1h,4h-hexafluorobutyloxy) phosphazene		186406-47-3
HP-1521	hexakis(1h,1h,5h-octafluoropentoxy) phosphazene		16059-16-8
HP-1821	hexakis(1h, 1h, 6h-decafluorohexyloxy) phosphazene		186406-48-4
HP-2121	hexakis(1h, 1h, 7h-dodecafluoroheptoxy) phosphazene		3830-74-8
HP-2421	hexakis(1h, 1h, 8h-tetradecafluorooxtylooxy) phosphazene		186406-49-5
HP-2721	hexakis(1h, 1h, 9h-perfluorononyloxy) phosphazene		186043-67-4
SDGRG	H-Ser-Asp-Gly-Arg-Gly-OH	Sigma-Aldrich (S3771)	108608-63-5
GRGDS	H-Gly-Arg-Gly-Asp-Ser-OH	Sigma-Aldrich (G4391)	96426-21-0
$GD1_a$	disialoganglioside GD_{1a} (d18:1/18:0)	Matreya, LLC (1062)	12707-58-3
$GD1_b$	disialoganglioside GD_{1b} (d18:1/18:0)	Matreya, LLC (1501)	19553-76-5
D-(+)-Melezitose, Mz	α-D-glucosyl-(1,3)-β-D-fructosyl-(2,1)-α-D-glucose	Alfa Aesar (AAB2220909)	207511-10-2
D-(+)-Raffinose, R	α-D-galactosyl-(1,6)-α-D-glucosyl-β-D-fructose	Waters Corp. (700004768)	512-69-6
Isomaltotriose, I	α-D-glucosyl-(1,6)-α-D-glucosyl-(1,6)-D-glucose	Sigma-Aldrich (I0381)	3371-50-4
Maltotriose, M	α-D-glucosyl-(1,4)-α-D-glucosyl-(1,4)-D-glucose	Sigma-Aldrich (M8378)	1109-28-0
TG 18:3/18:3/18:3 (cis 6,9,12)	1,2,3-Tri-γ-linolenoyl-*rac*-glycerol	Cayman Chemical (10009825)	60756-74-3
TG 18:3/18:3/18:3 (cis 9,12,15)	1,2,3-Tri-α-linolenoyl-*rac*-glycerol	Cayman Chemical (10009758)	14465-68-0

Table S2. Corresponding Wave Speeds for SLIM IM Wave Switching Frequencies**.**

SLIM IM TW Output Drive Frequency (kHz)	Equivalent Wave Speed (m/s) [a.]
5	45
10	90
15	135
20	180
25	225

a. To calculate the wave speed, the drive frequency (in kHz) is multiplied by the pad-to-pad distance (1.125 mm) and the number of pads traversed to complete one phase of the wave form (8 pads).

Table S3. The Second Highest Resolving Powers Observed

Tune Mix Ion	Highest R_p Measured (CCS/ΔCCS) [a.]	Corresponding Parameter		Corresponding Arrival Time (ms)
		Wave Amplitude (V_{pp})	Wave Speed (m/s)	
m/z 622	283.7 ± 20.7 (3)	40	180	215.0
m/z 922	258.7 ± 8.0 (3)	40	225	437.9
m/z 1222	247.1 ± 9.7 (3)	40	225	601.2
m/z 1522	243.0 ± 7.1 (2)	35	90	330.8
m/z 1822	255.1 ± 7.1 (3)	35	90	426.5
m/z 2122	257.9 ± 12.7 (3)	35	90	523.8
m/z 2422	268.8 ± 8.9 (3)	40	90	458.2
m/z 2722	253.0 ± 10.1 (3)	35	90	721.8

a. Highest R_p is averaged over replicate measurements, denoted in parenthesis. The time-to-CCS conversion is determined from Equation 3 in the main text using the differences between the tune mix ion and the next highest m/z ion in the spectrum.

Table S4. CCS Measurements from DTIM

<table>
<tr><th>Analyte Abbreviation</th><th>Peak</th><th>Ion Form</th><th>Ion Mass (m/z)</th><th>$^{DT}CCS_{N2}$ [a.] ($Å^2$)</th><th colspan="2">CCS difference</th></tr>
<tr><td>SDGRG</td><td>1</td><td rowspan="2">$[M+H]^+$</td><td rowspan="2">491.1970</td><td>203.5±0.4 (5)</td><td colspan="2" rowspan="2">0.9%</td></tr>
<tr><td>GRGDS</td><td>2</td><td>205.4±0.4 (5)</td></tr>
<tr><td>TG 18:3/18:3/18:3 (cis 9,12, 15)</td><td>1</td><td rowspan="2">$[M+Na]^+$</td><td rowspan="2">895.6791</td><td>313.3±0.4 (5)</td><td colspan="2" rowspan="2">0.6%</td></tr>
<tr><td>TG 18:3/18:3/18:3 (cis 6, 9,12)</td><td>2</td><td>315.3±0.5 (5)</td></tr>
<tr><td>D-(+)-Melezitose, Mz</td><td>1</td><td rowspan="4">$[M+Na]^+$</td><td rowspan="4">527.1582</td><td>201.8±0.4 (5)</td><td rowspan="2">3.4%</td><td>--</td></tr>
<tr><td>D-(+)-Raffinose, R</td><td>2</td><td>208.8±0.4 (5)</td><td rowspan="2">1.0%</td></tr>
<tr><td>Isomaltotriose, I</td><td>3</td><td>210.9±0.4 (5)</td><td rowspan="2">0.9%</td></tr>
<tr><td>Maltotriose, M</td><td>4</td><td>212.9±0.4 (5)</td><td>--</td></tr>
<tr><td>$GD1_b$</td><td>1</td><td rowspan="2">$[M-2H]^{-2}$</td><td rowspan="2">917.4788</td><td>446.2±0.8 (5)</td><td colspan="2" rowspan="2">0.7%</td></tr>
<tr><td>$GD1_a$</td><td>2</td><td>449.1±0.8 (5)</td></tr>
<tr><td>$GD1_b$</td><td>1</td><td rowspan="2">$[M+2Na]^{+2}$</td><td rowspan="2">941.4753</td><td>446.3±0.8 (5)</td><td colspan="2" rowspan="2">1.9%</td></tr>
<tr><td>$GD1_a$</td><td>2</td><td>454.6±0.8 (5)</td></tr>
<tr><td colspan="7">a. CCS measurements obtained from a drift tube (DT) using a standardized CCS acquisition method. Number of replicates are noted in parenthesis.</td></tr>
</table>

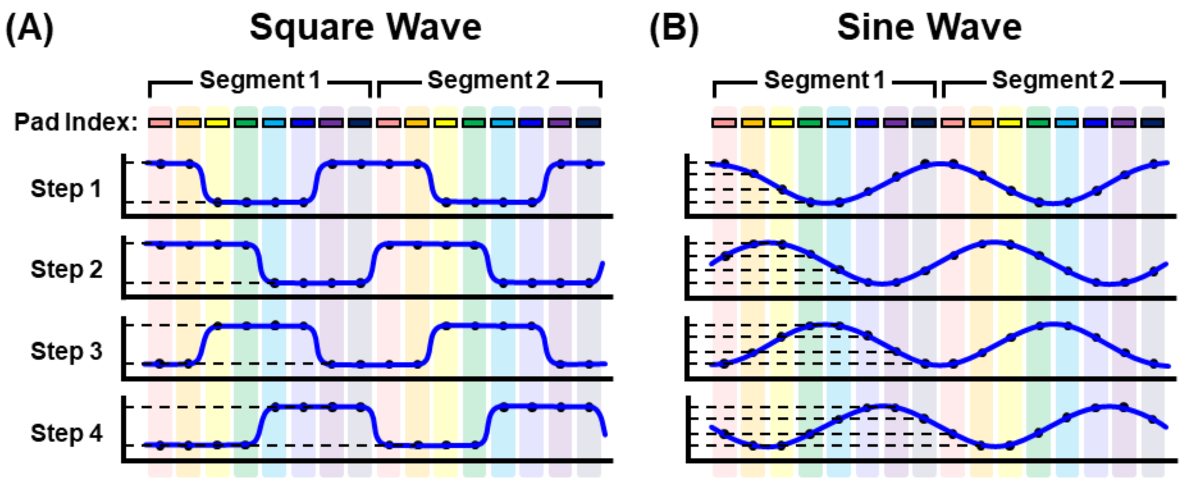

Figure S1 – Traveling wave operation of SLIM IM illustrated for two SLIM segments (8 pads each) using **(A)** a square waveform, and **(B)** a sine waveform.

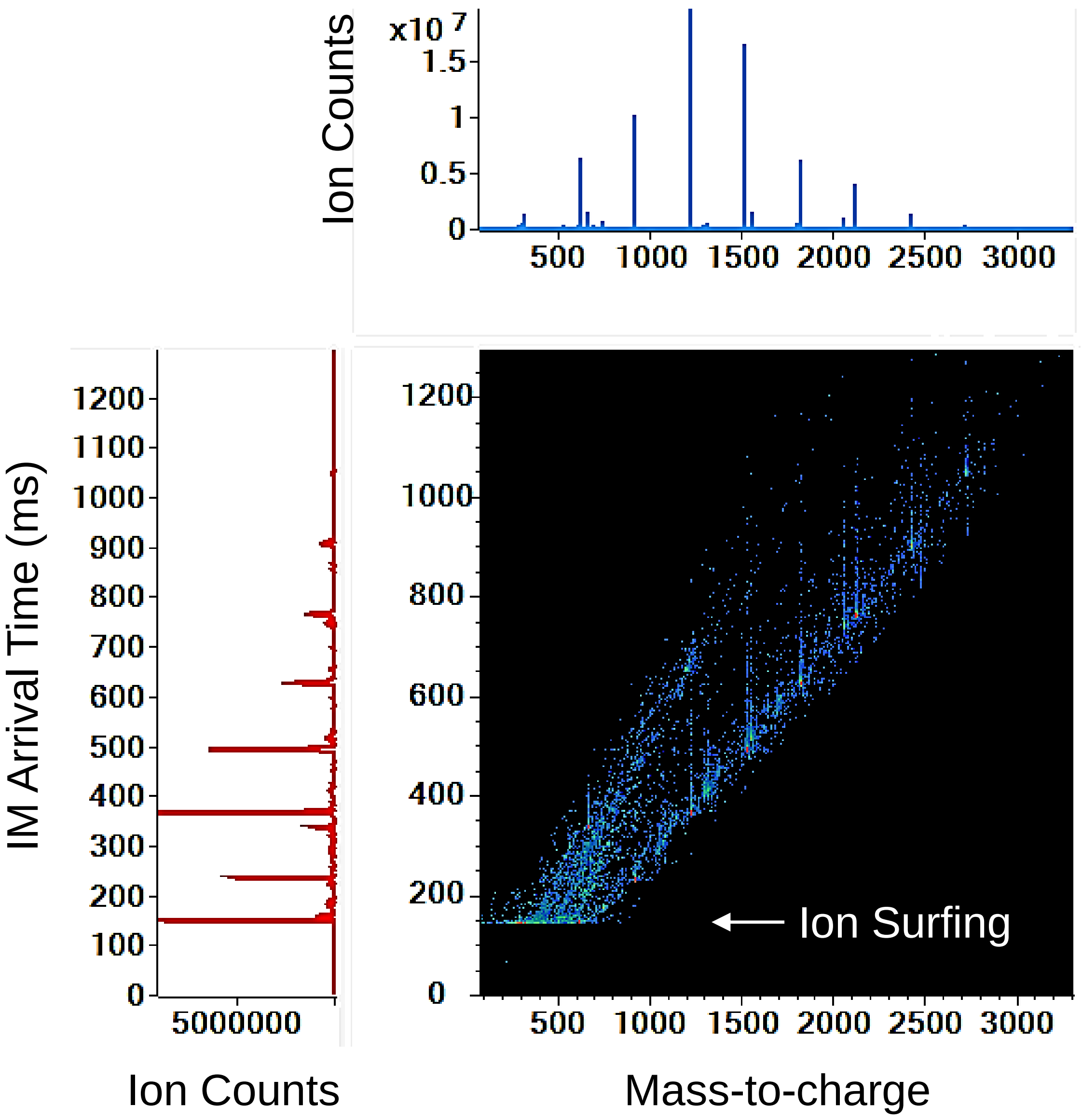

Figure S2 – A multidimensional IM-MS spectrum of tune mix ions illustrating the spectrum observed under conditions where the lower m/z ions are "surfing" the traveling wave and no IM separation occurs. Here, SLIM conditions were 90 m/s and 30 V_{pp}. Under surfing conditions, all ions exhibit the same arrival times, which corresponds to the wave propagation time through the SLIM device (90 m/s / 13 m = ~144 ms).

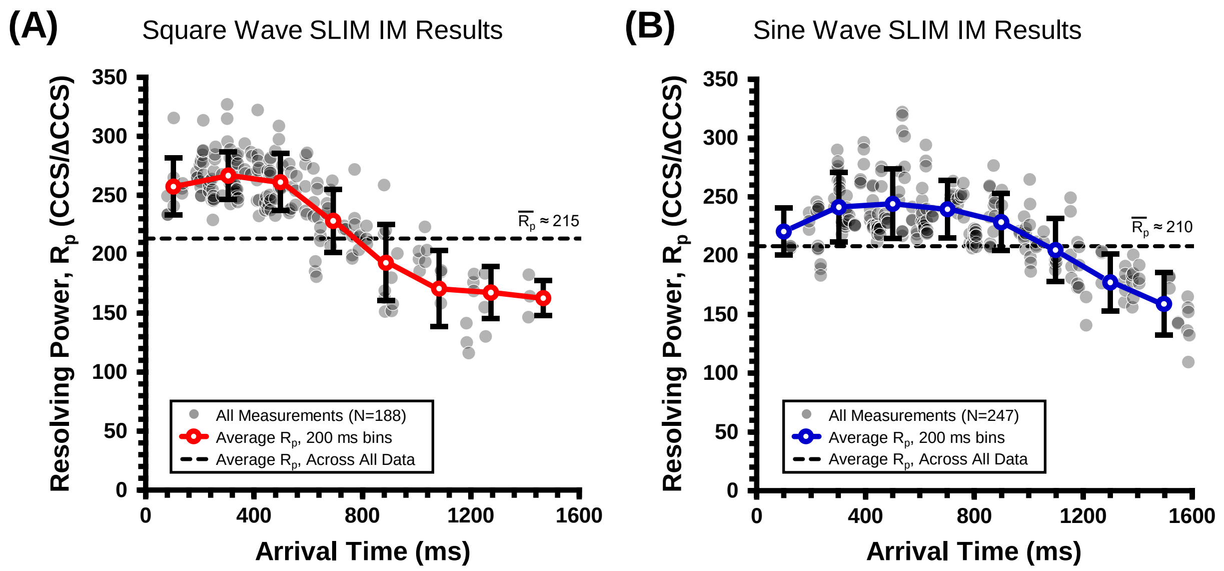

Figure S3 – Plots projecting the single-peak resolving power in CCS-space, R_p(CCS), as a function of the measured arrival times for **(A)** square wave, and **(B)** sine wave operation of the traveling wave. Plots contain data points for all wave amplitudes (30, 35, and 40 V_{pp}) and wave speeds (45, 90, 135, 180, and 225 m/s) surveyed in these experiments, with data points omitted for ions which do not exhibit IM-selective behavior (that is, ions which "surf" the wave and thus are not resolved). For square wave, 52,2% of the measurements are IM-selective (188 out of 360), whereas for sine wave, 68.6% of the ions (247 out of 360) are observed to separate via the traveling wave. In both projections, the circle markers connected with solid traces represent the average R_p(CCS), determined for 200 ms arrival time bins, and the horizontal dotted line corresponds to the average R_p(CCS) across the entire dataset. Whereas both datasets exhibit similar resolving powers, the square wave data (panel A) transmits fewer ions under IM-selective conditions across the parameters surveyed.

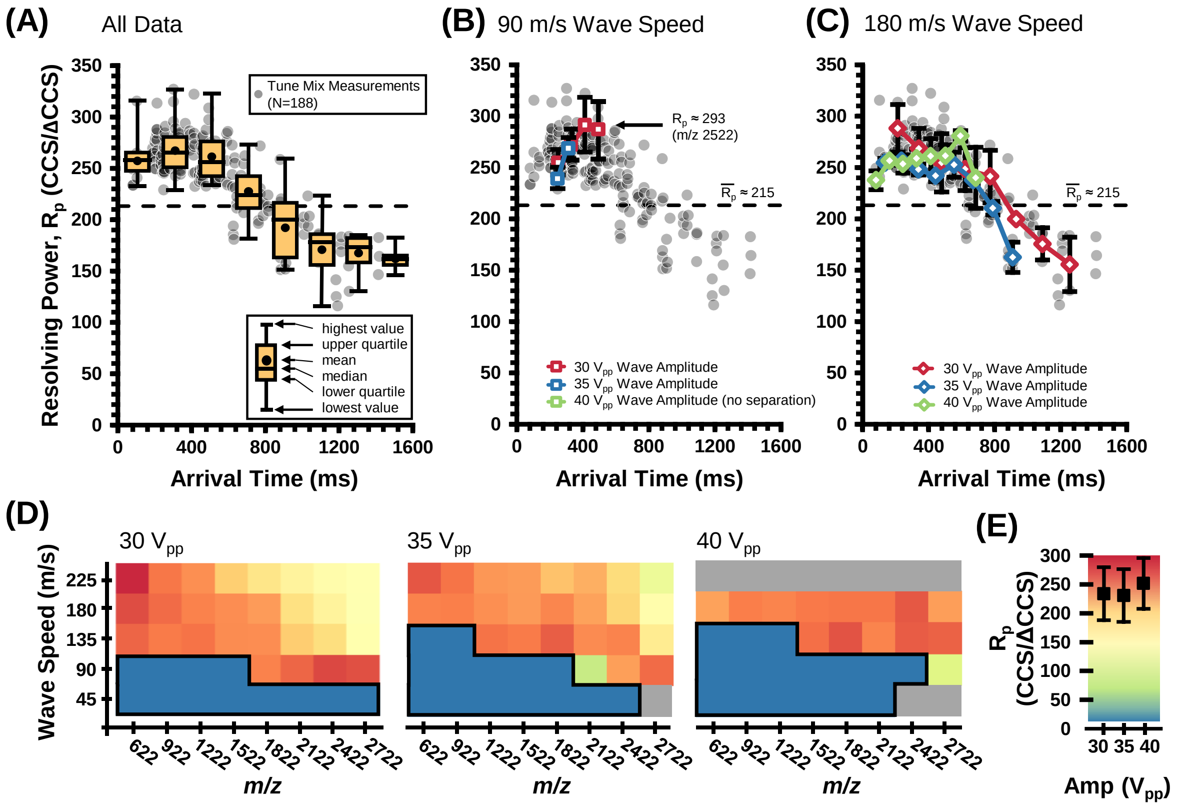

Figure S4 – Plots assessing the resolving power performance for a square waveform under the traveling wave parameters surveyed in this work. **(A)** CCS-based R_p values (N=188) as a function of the measured arrival time for tune mix components (cyclophosphazenes) measured across various SLIM IM parameters, including wave speeds (45, 90, 135, 180, and 225 m/s) and wave amplitudes (30, 35, and 40 Vpp). Box and whisker overlays summarize the data within 200 ms bins. **(B)** Average R_p(CCS) values (3 replicates per data point) determined for 90 m/s wave speed at 30, 35, and 40 V_{pp} wave amplitudes. Under the highest amplitude evaluated (40 V_{pp}), no ions are transmitted under IM-selective arrival times for square wave. **(C)** Average R_p(CCS) values calculated for 180 m/s data. The horizontal dotted line in these scatter plots represents the average R_p across the entire dataset (ca. 215). **(D)** Heat maps visualizing the resolving powers for each tune mix component (x-axis) at each wave speed (y-axis) for wave amplitudes of 30 (left panel), 35 (middle panel), and 40 V_{pp} (right panel). Each square represents an average of three replicate measurements. Here, dark blue squares represent conditions in which ions are transmitted, but no IM separation occurs (i.e., ion "surfing" conditions), whereas grey boxes correspond to conditions where ion transmission is poor. **(E)** Color scale for panel D with average and standard deviation values overlaid.

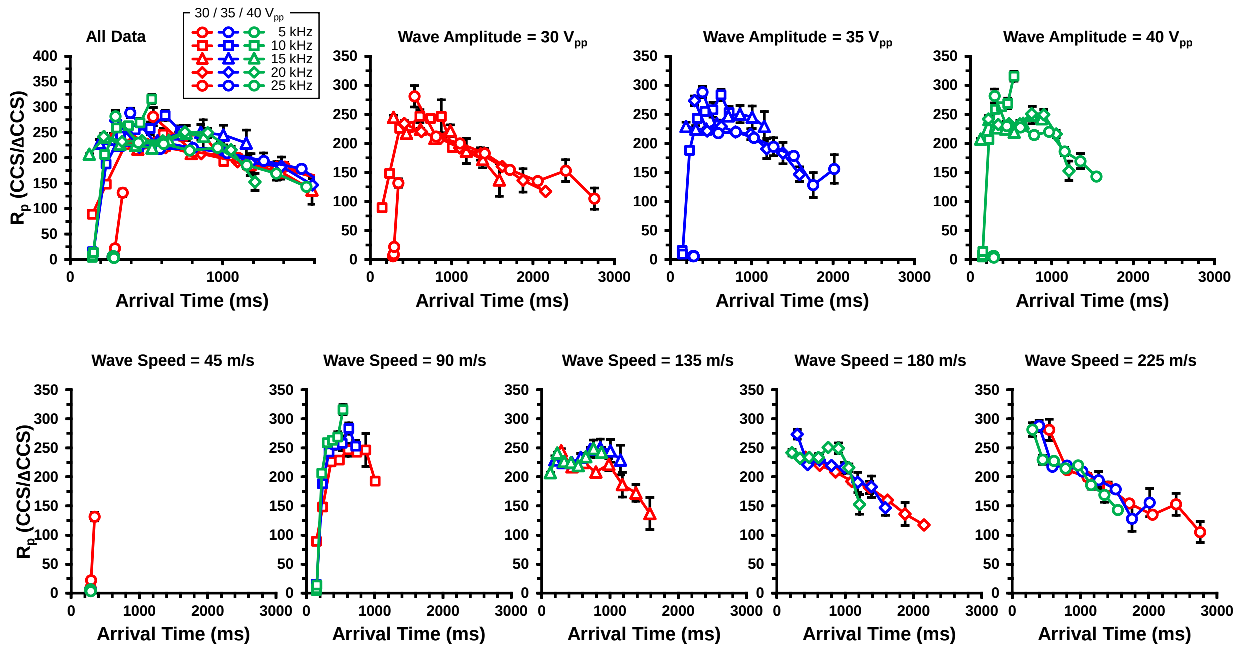

Figure S5 – Resolving power (CCS/ΔCCS) results for all tune mix ions, separated into the different wave amplitudes and wave speeds surveyed. Here, ions which are not fully separated by the traveling waves (surfing ions) are also included.

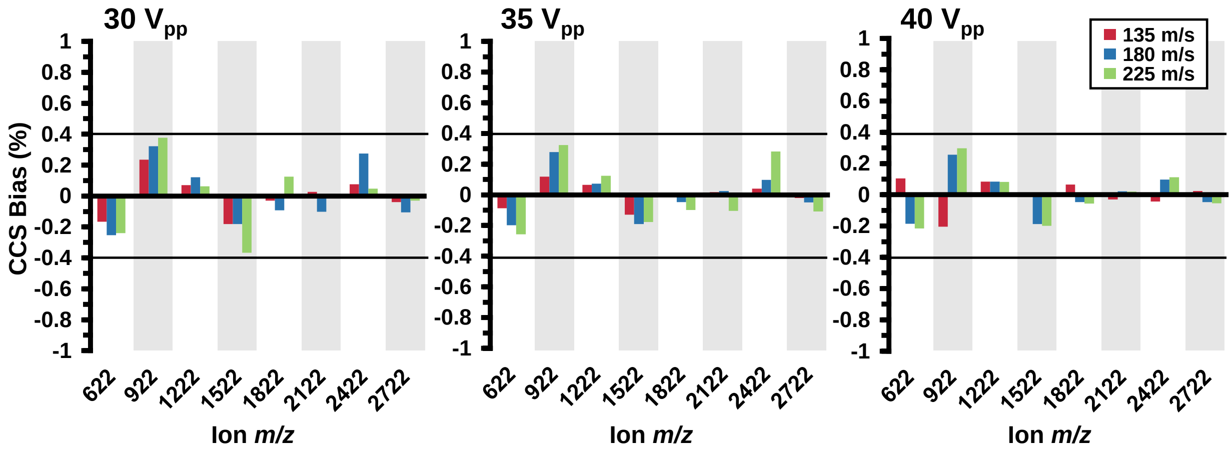

Figure S6 – CCS calibration biases observed across all SLIM IM parameters for a 3rd order polynomial fit. Dotted lines represent ±0.4% bias of the calibrated CCS compared with the reference CCS values.

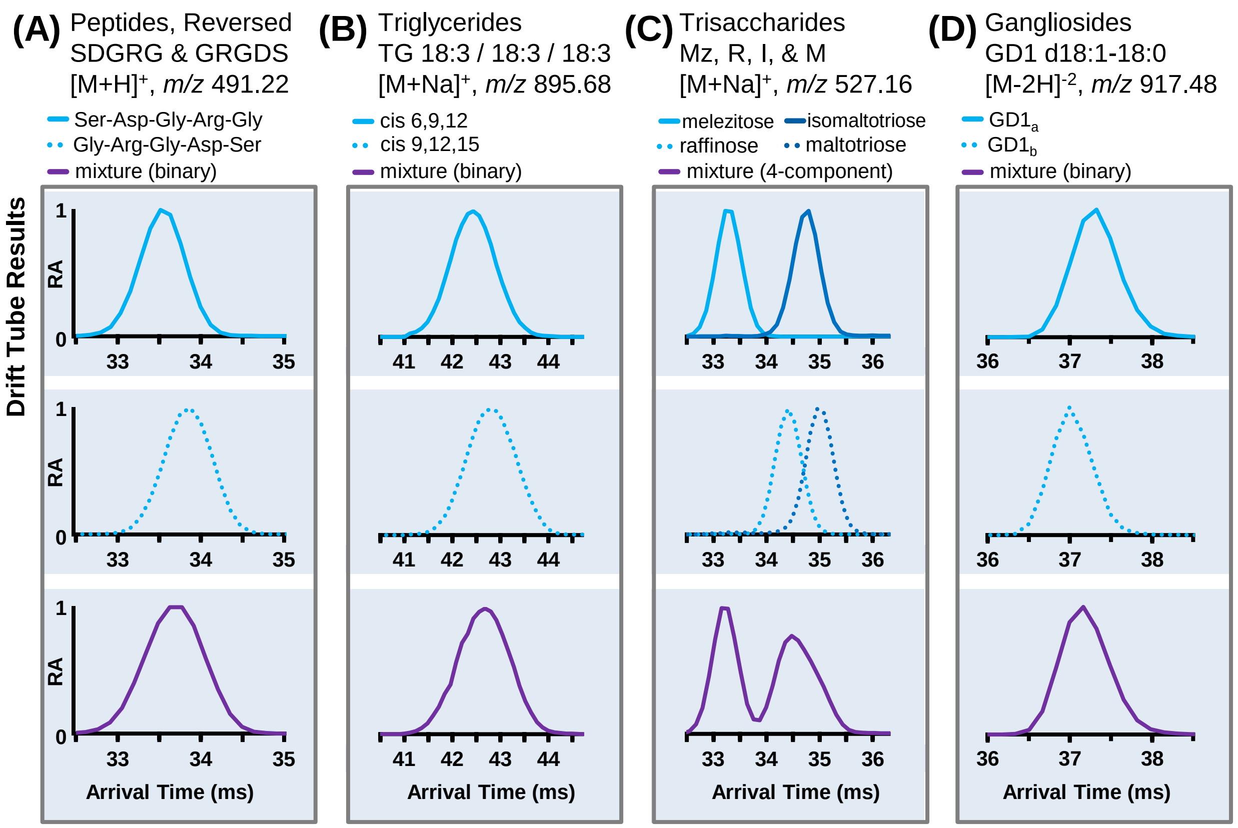

Figure S7 – Drift tube ion mobility traces for the individual isomer standards and their mixtures, delineated into separate plots for clarity.

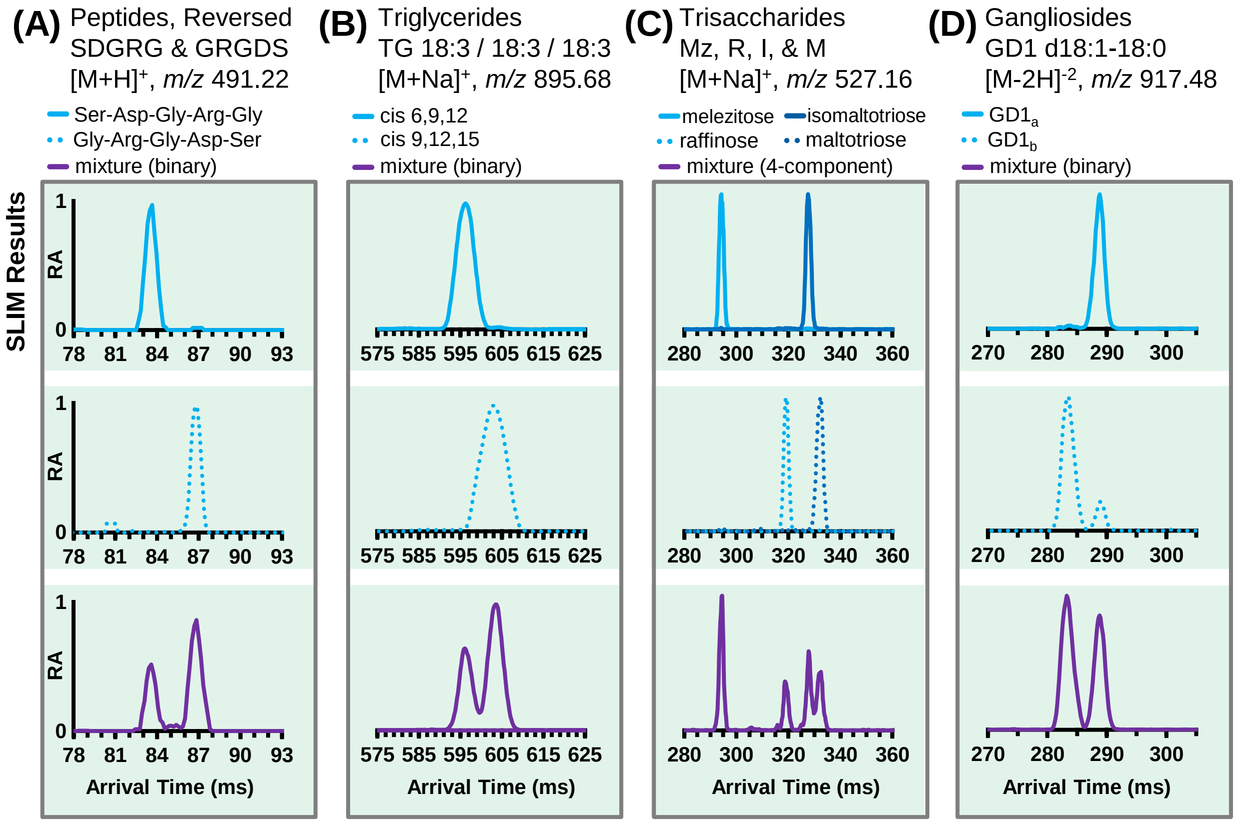

Figure S8 – SLIM ion mobility traces for the individual isomer standards and their mixtures, delineated into separate plots for clarity.
